# Immediate-Early Genes Modulation by Antipsychotics: Translational Implications for a Putative Gateway to Drug-Induced Long-Term Brain Changes

**DOI:** 10.3389/fnbeh.2017.00240

**Published:** 2017-12-11

**Authors:** Andrea de Bartolomeis, Elisabetta F. Buonaguro, Gianmarco Latte, Rodolfo Rossi, Federica Marmo, Felice Iasevoli, Carmine Tomasetti

**Affiliations:** Laboratory of Molecular and Translational Psychiatry and Unit of Treatment Resistant Psychosis, Section of Psychiatry, Department of Neuroscience, Reproductive Sciences and Odontostomatology, University School of Medicine “Federico II”, Naples, Italy

**Keywords:** Arc, BDNF, Homer1a, clozapine, haloperidol, schizophrenia, bipolar disorders, cognition

## Abstract

An increasing amount of research aims at recognizing the molecular mechanisms involved in long-lasting brain architectural changes induced by antipsychotic treatments. Although both structural and functional modifications have been identified following acute antipsychotic administration in humans, currently there is scarce knowledge on the enduring consequences of these acute changes. New insights in immediate-early genes (IEGs) modulation following acute or chronic antipsychotic administration may help to fill the gap between primary molecular response and putative long-term changes. Moreover, a critical appraisal of the spatial and temporal patterns of IEGs expression may shed light on the functional “signature” of antipsychotics, such as the propensity to induce motor side effects, the potential neurobiological mechanisms underlying the differences between antipsychotics beyond D2 dopamine receptor affinity, as well as the relevant effects of brain region-specificity in their mechanisms of action. The interest for brain IEGs modulation after antipsychotic treatments has been revitalized by breakthrough findings such as the role of early genes in schizophrenia pathophysiology, the involvement of IEGs in epigenetic mechanisms relevant for cognition, and in neuronal mapping by means of IEGs expression profiling. Here we critically review the evidence on the differential modulation of IEGs by antipsychotics, highlighting the association between IEGs expression and neuroplasticity changes in brain regions impacted by antipsychotics, trying to elucidate the molecular mechanisms underpinning the effects of this class of drugs on psychotic, cognitive and behavioral symptoms.

## Introduction

There is growing interest in unraveling the cellular mechanisms putatively involved in long-term changes in brain architecture and function following antipsychotic administration (Ahmed et al., 2008; Ho et al., 2011; Cannon et al., 2015; Vita et al., 2015; Yue et al., 2016; Emsley et al., 2017). *In vivo* human studies have pointed out that volumetric and functional changes may be detected after acute antipsychotic treatments (Emsley et al., 2015, 2017). However, the long-term consequences of these acute changes remain still elusive.

Immediate-early genes (IEGs) may represent a significant candidate to explore how acute antipsychotics administration may set the molecular scenario for long-term changes.

**Figure 1:**
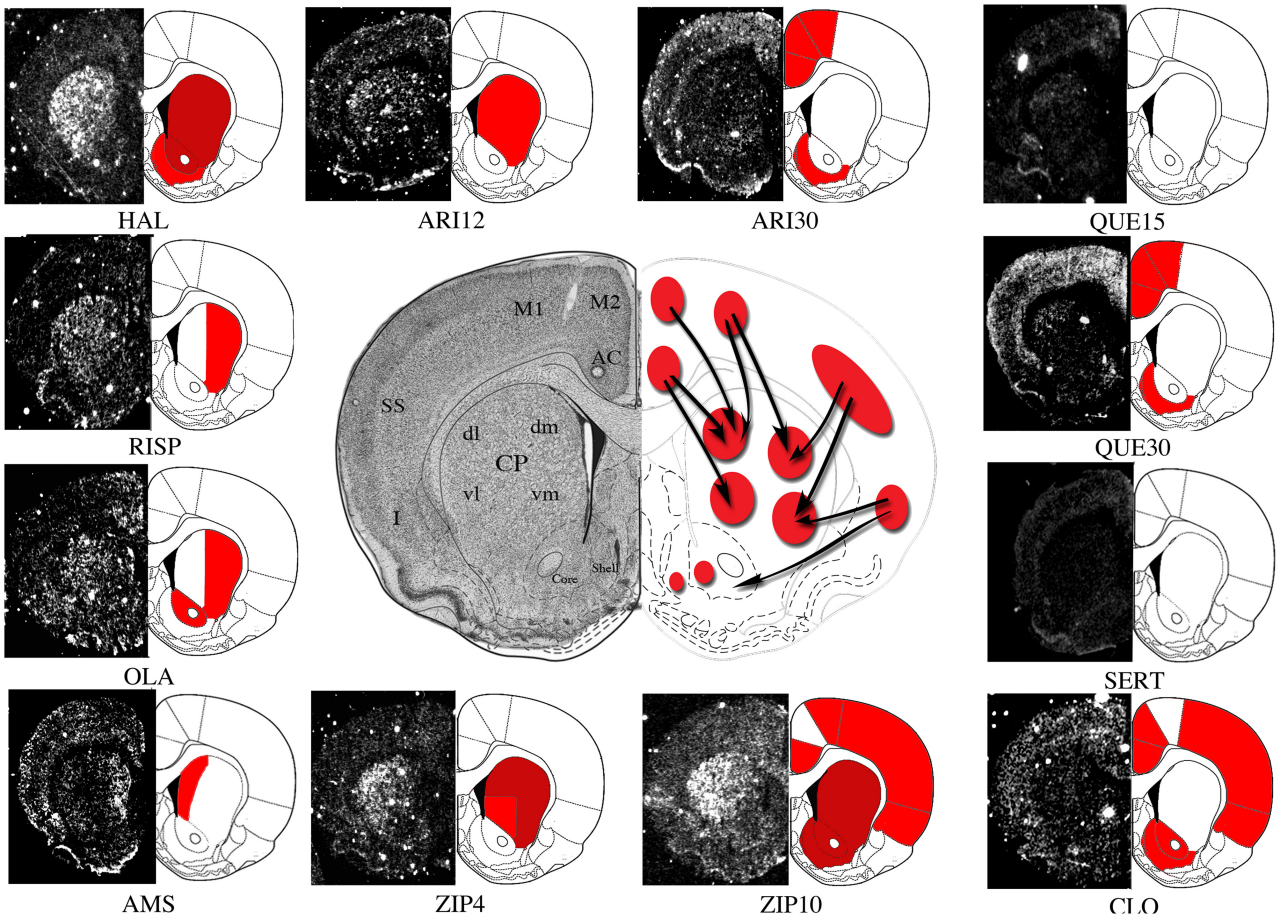
Representative molecular imaging of *Homer1a* IEG expression by acute antipsychotics administration. Molecular imaging of IEGs expression may represent a tool to investigate topographic distribution of antipsychotic-mediated acute and long-term molecular effects within brain Regions of Interest (ROIs). Here we show a representative depiction of *Homer1a* IEG expression by different antipsychotics acutely administered in rodents. The autoradiographic film images of *Homer1a* mRNA detected by means of *in situ* hybridization histochemistry in coronal brain sections have been extracted from different studies carried on by our laboratory (Polese et al., 2002; Ambesi-Impiombato et al., 2007; Tomasetti et al., 2007, 2011; Iasevoli et al., 2009, 2010a,b, 2011; De Bartolomeis et al., 2015a) and representatively placed side by side in order to outline gene expression topography after treatments with haloperidol (HAL), olanzapine (OLA), sertindole (SERT), amisulpiride (AMS), risperidone (RISP), clozapine (CLO), and different doses of ziprasidone (4 mg/kg, ZIP4; 10 mg/kg, ZIP10), aripriprazole (12 mg/kg, ARI12; 30 mg/kg, ARI30), quetiapine (15 mg/kg, QUE15; 30 mg/kg, QUE30). *Homer1a* is a postsynaptic effector of plastic synaptic changes mainly mediated by dopamine and glutamate-dependent signaling pathways. Therefore, in this case, IEG molecular imaging may also provide putative information on antipsychotic-triggered changes in synaptic plasticity. ROIs: AC, Anterior Cingulate Cortex; M2, Medial Agranular Cortex; M1, Motor Cortex; SS, Somatosensory Cortex; I, Insular Cortex; dmCP, Dorso Medial Caudate-Putamen; dlCP, Dorso Lateral Caudate-Putamen; vlCP, Ventro Lateral Caudate-Putamen; vmCP, Ventro Medial Caudate-Putamen; Core, Nucleus Accumbens; Core, Shell, Nucleus Accumbens, Shell. Red, significant gene induction as compared to the respective control (*p* < 0.05); Dark red, significant gene induction as compared to the respective control (*p* < 0.001).

New insights in IEGs expression following acute or chronic antipsychotic administration in preclinical models may help to fill the gap between primary molecular responses to antipsychotic administration and putative long-term synaptic changes (Figure 1). Recent observations are opening new avenues in our understanding of how antipsychotics work and strongly challenge the old idea that significant changes in synaptic plasticity may be caused by prolonged treatments. Indeed, multiple lines of evidence demonstrated that *in vivo* antipsychotic treatment may significantly impact the architecture of the synapse, as well as the re-arrangement of gene expression of scaffolding and adaptor proteins after acute exposure to the drugs. In line with this view, haloperidol acute administration has been shown to reduce dendritic spines size, possibly through a beta-adducin-mediated mechanism (Engmann et al., 2016). Moreover, the acute administration of typical and atypical antipsychotics has been demonstrated to re-arrange the topography of *Homer1a* gene expression in cortical and sub-cortical brain regions (Buonaguro et al., 2017b).

Acute antipsychotics administration has been demonstrated to impact signal-transduction pathways in specific brain regions with significant implications for long-term treatment (De Bartolomeis et al., 2013b, 2015a). For instance, acute i.v. infusion at therapeutic doses of haloperidol may trigger changes in the volume of the striatum (Tost et al., 2010), this effect being consistent with the rapid and transient IEGs induction by acute dopamine D2 receptors (D2Rs) blockade.

IEGs activation after antipsychotics acute administration could be pivotal to dissect primary molecular and cellular events that may prime the long-term effects of antipsychotic treatment. Similarly, multiple studies have pointed out the significant functional changes in cortical and subcortical networks after acute administration of antipsychotics (Emsley et al., 2015, 2017).

At the same time, new exciting discoveries in early gene functions have reinvigorated the research on the role of IEGs in the brain, thanks also to novel techniques, such as the following: serial two-photon tomography (STP) for automated whole-brain histology using fluorescent reporters (Ragan et al., 2012); light sheet fluorescence microscopy (LSFM) coupled with tissue clearing for imaging IEG expression in the intact brain (Renier et al., 2016); optogenetics for selectively activate target neurons (Bepari et al., 2012).

Despite the relevance of the issue, the role of IEGs in antipsychotics action has not been reviewed recently and a comprehensive analysis is still lacking.

Herein, starting from the major IEGs proven to be induced by antipsychotics and from their involvement in brain functions believed to be translationally relevant for schizophrenia as well as for antipsychotic mechanism of action, we will review the following issues:

1. IEGs regulation with focus on dopamine-related mechanisms relevant for or related to antipsychotics action;
2. IEGs expression in psychosis and differential modulation by antipsychotics.

Moreover, we will consider:

1. How IEGs induction may impact directly or indirectly synaptic architecture;
2. How IEGs are differentially affected by acute and chronic antipsychotic treatment;
3. How antipsychotics with different receptor profile or the same antipsychotic at different doses may affect the expression of different IEGs with regard of brain topography.

## Background: antipsychotics, IEGs, and brain changes

### IEGs relevance for synaptic plasticity

IEGs are a heterogeneous class of genes that are rapidly and transiently activated by a large number of stimuli, including environmental (i.e., light/dark phase changes, exposure to behavioral stressors such as intruder animals, learning session during acquisition tasks), pharmacological, and physical stimuli (Perez-Cadahia et al., 2011; Sauvage et al., 2013). IEGs represent a primary response to cellular perturbation, which is a standing process that is activated at the transcriptional level and occurs in the absence of *de novo* protein synthesis. IEGs are dynamically regulated by different forms of synaptic activity underlying information processing and storage, therefore they are excellent candidates involved in both Hebbian and homeostatic plasticity (Hu et al., 2010; Hayashi et al., 2012; Shin et al., 2012). Several studies demonstrated, indeed, that long-term forms of synaptic plasticity—such as long-term potentiation (LTP)—require new production of intracellular macromolecules, whereas short-term synaptic plasticity processes do not (Kandel, 2001; Hayashi et al., 2012). IEGs expression occurring promptly after stimuli is considered a fundamental step for the establishment of synaptic plasticity, since synaptic plasticity changes may be prevented when mRNA synthesis is blocked early after the induction of a stimulus (Lanahan and Worley, 1998). Thus, IEGs may be considered as “gateway” genes controlling synaptic plasticity and may underlie processes like learning and memory formation.

IEGs encode a large number of proteins with different functions, such as transcription factors (e.g., c-Fos, Egr1, NGFI-B), postsynaptic proteins (e.g., Norbin, Homer 1a, Arc) and signaling molecules (e.g., RSG2, CaMKII).

The induction of an IEG is one of the earliest intracellular mechanism mediating the cellular response to external stimuli (Lanahan and Worley, 1998). According to these view, IEGs induction may be considered a recent activity marker, and its assessment may be used to determine when specific neural populations are activated, making possible to assess the extent of antipsychotics spatial and temporal impact on neural plasticity in different brain areas.

### IEGs: a putative gateway for antipsychotic-induced brain changes

Antipsychotic drugs are the mainstay of pharmacological treatment for schizophrenia, and their use has been expanded for the treatment of bipolar disorder and, in some cases, for pervasive disorders of the autistic spectrum (Geddes and Miklowitz, 2013). All antipsychotics share a variable degree of antagonism, or partial agonism, at D2 dopamine receptors (D2Rs) and both therapeutic and motor side effects of either typical or atypical antipsychotic drugs have been proven to directly depend on the occupancy of D2Rs (Seeman, 2002; Ginovart and Kapur, 2012). However, besides the dynamics of D2Rs binding by different antipsychotics, emerging evidence demonstrates that the study of the downstream signaling elicited by these compounds may help to better understand the mechanisms of action implicated in their clinical effects (De Bartolomeis et al., 2013a; Iasevoli et al., 2013). Additionally, dissecting the molecular basis of antipsychotic actions may shed light on new avenues of investigation to bypass the critical issues related to receptor pharmacodynamics, such as D2Rs down- or up-regulation and D2Rs supersensitivity, which have been considered among the potential reasons of antipsychotic treatment resistance (Seeman, 2002; Nnadi and Malhotra, 2007; Seeman and Seeman, 2014; Oda et al., 2015).

In the last decades, several studies on the effects of typical and atypical antipsychotics on brain IEGs expression have been carried out, trying to unravel the bases of regional neuronal response to pharmacological stimuli (Dragunow, 1990; Miller, 1990; Young et al., 1998; Semba et al., 1999; Beaudry et al., 2000; Kovacs et al., 2001; Cochran et al., 2002), as well as to shed light on the molecular mechanisms implicated in antipsychotic actions (Deutch et al., 1991, 1995). Transcriptional fingerprint of IEGs, and their functionally related molecules, has progressively emerged as a potential methodology to explore temporal and functional brain regions recruitment by antipsychotics and psychotomimetic compounds (Gonzalez-Maeso et al., 2003; Tomasetti et al., 2007; Sakuma et al., 2015). Moreover, IEGs have been found to be modulated also by other psychotropic drugs, such as antidepressants (De Foubert et al., 2004; Alme et al., 2007; Molteni et al., 2008; Calabrese et al., 2011), mood stabilizers (De Bartolomeis et al., 2012), as well as by the combination of antipsychotics and antidepressants or mood stabilizers, which has been demonstrated to differentially induce IEGs patterns of expression as compared to the compounds when individually administered (Dell'aversano et al., 2009; Tomasetti et al., 2011).

## Literature research methodology

As a first step, we carried out multiple searches on Pubmed, Scopus, and ISI Web of Knowledge using as a reference the following keywords (we reported in parentheses the results obtained on Pubmed for the search conducted on May 2017): Immediate Early Genes AND brain (2335); Immediate Early Genes AND antipsychotics (84); Immediate early genes AND antipsychotics AND brain (74). Successively, we searched by the name of each single IEG or related gene of interest, together with the keywords “brain” AND/OR “antipsychotics.” The name of the IEGs or related genes searched were: Arc/Arg, BDNF, c-fos, fos, c-Jun, jun, Egr1, Delta-fos, Narp1, NPAS-4, Homer1, Homer2, Homer3, Nor1, Nurr, Nurr1, NGFI-B/Nur77, Nerve Growth Factor Inducible-B, NR4A.

A “parallel search” was conducted using as key words the combination the following ones: antipsychotics AND acute effects, antipsychotics AND brain volume, antipsychotics AND cortical thickness, antipsychotics AND acute AND PET, antipsychotics AND acute AND fMRI, antipsychotics AND brain changes.

For the above-mentioned first search (keywords: Immediate Early Genes AND brain), each abstract retrieved was considered for coherence of the subject with the content of the review by two independent co-authors. If the text of the abstract was coherent with the review, the full text was considered and the references double-checked for potential new articles of relevance. All the articles retrieved with the second search (Immediate-early genes AND antipsychotics) and the third search (Immediate-early genes AND antipsychotics AND brain), as well as the articles retrieved by the search for single early gene name, were considered for the full text. The results of the first search where then compared with the result of the other searches and with the one of the “parallel search.”

## C-*fos*, Δ*FosB*, C-*Jun*: mapping the neural activity in response to antipsychotics, old and new findings

Synaptic plasticity processes occurring in response to neural activity are mediated by complex programs of gene expression controlled by transcription factors (TFs; Beckervordersandforth et al., 2015; Ortega-Martinez, 2015; Ehrlich and Josselyn, 2016).

**Figure 2:**
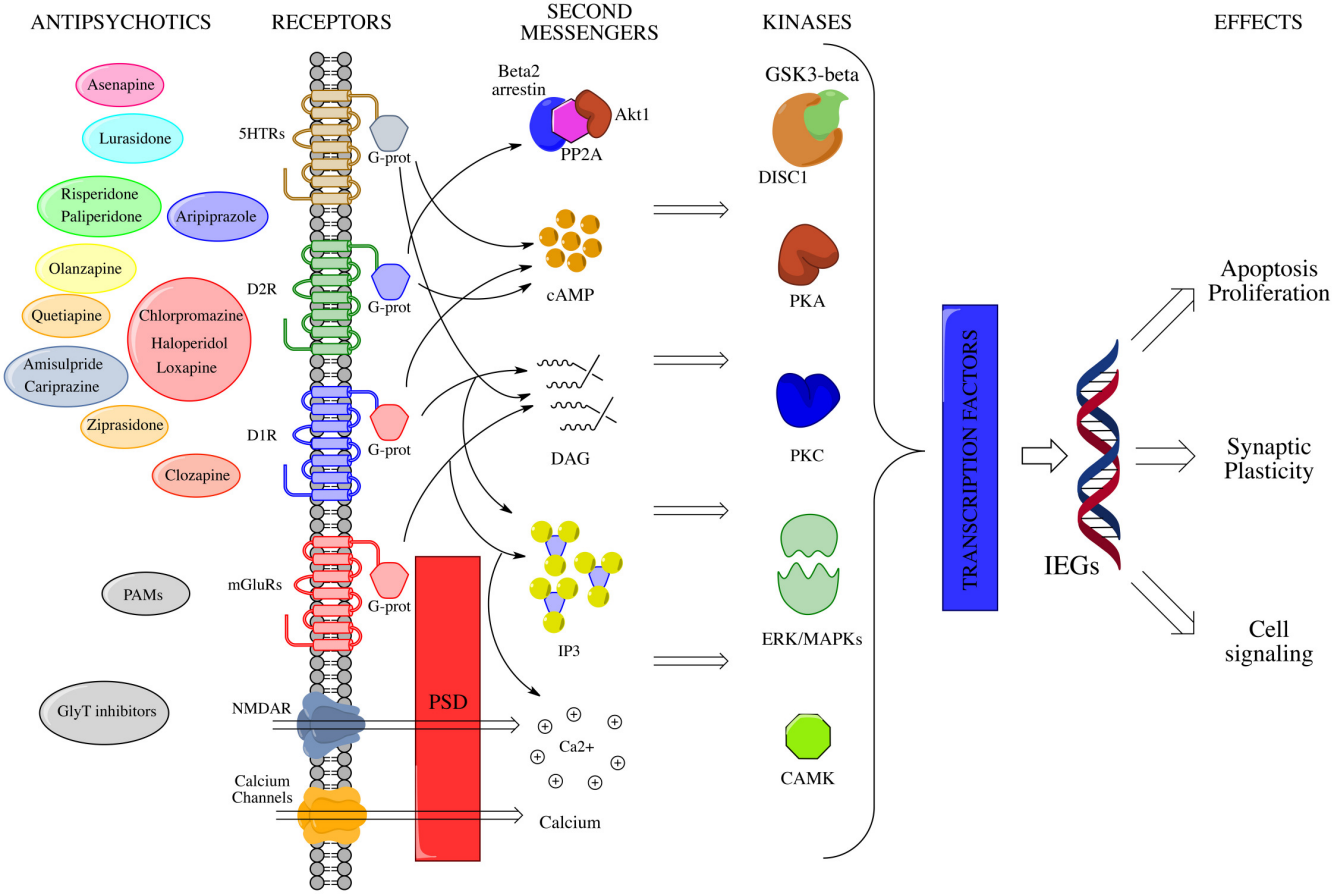
IEGs modulation by antipsychotic drugs. Membrane receptors activate multiple signal transduction pathways, which interact at several sites through the mediation of a large number of second messengers. A crucial role in the post-membrane interaction is played by the PSD, which serves as a physical connection among ionotropic and metabotropic glutamate receptors, and links them to intracellular calcium stores. All these pathways converge in the end to appropriate nuclear targets (i.e., transcription factors, TFs) via specific effectors, largely kinases (e.g., CAMK, MAPKs, PKA etc.), in order to fine modulate long-term activity dependent neuronal rearrangements through changes in IEGs expression levels. On the left side of the picture, antipsychotic compounds are depicted near to the membrane receptors to which they show maximum affinity. NMDAR, N-methyl-D-aspartate glutamate receptor; mGluR1a/5, metabotropic glutamate receptor type 1a/5; D1, dopamine receptor D1; D2, dopamine receptor D2; DAG, diacylglycerol; IP3, inositol 1,4,5-trisphosphate; Akt1, RAC-alpha serine/threonine-protein kinase; PP2A, protein phosphatase 2A; GSK3b, Glycogen synthase kinase 3 beta; DISC1, Disrupted in schizophrenia 1; CAMK, Ca^2+^/calmodulin-dependent protein kinase; cAMP, cyclic adenosine monophosphate; PKC, protein kinase C; PKA, protein kinase A; MAPKs, mitogen-activated protein kinases; ERK, extracellular signal-regulated kinase; CREB-P, cAMP response element-binding protein; IEGs, immediate early genes; PSD, post-synaptic density; PAMs, positive allosteric modulators of mGluRs; GlyT, glycine transporter.

Antipsychotics have been demonstrated to differentially impact IEGs encoding neural TFs, thus inducing a significant reprogramming in the expression of genes involved in synaptic plasticity (Figure 2; Table 1).

**Table 1:** Detection of IEGs expression evaluation in rodents after antipsychotics administration.

**Gene**	**Drug**	**Effect on gene expression**	**Brain region**	**References**
***c-fos***	Acute **Amisulpride**	↑	Medial Striatum	De Bartolomeis et al., 2013b
	Acute High dose **Asenapine**	↑	Striatum, NAc	De Bartolomeis et al., 2015a
	Acute **Clozapine**	↑	NAc, Thalamus, Striatum	Robbins et al., 2008
	Acute **Clozapine**	↑	NAc Shell	Werme et al., 2000; Polese et al., 2002
	Acute **Haloperidol**	↑	NAc Shell and Core, Medial, and Lateral Caudate Putamen, Lateral septum	Robertson et al., 1994; Werme et al., 2000 Robbins et al., 2008 Polese et al., 2002 De Bartolomeis et al., 2015a
	Acute **Haloperidol** or acute **Clozapine**	↑	Anteroventral Thalamus	Cochran et al., 2002
	Acute but not chronic **Risperidone**	↑	Striatum	Robinet et al., 2001
	Transient treatment with **Haloperidol** but not continuous treatment	↑	Striatum	Samaha et al., 2008
	Chronic **Clozapine** (after 6 days of washout)	↑	PFC, FC, NAc Core	Kontkanen et al., 2002
***c-jun***	Acute **Clozapine**	↑	NAc	Robbins et al., 2008
		↓	Hippocampus	
	Acute **Haloperidol**	↓	NAc	Robbins et al., 2008
	Chronic **Clozapine**	↑	FC, NAc Shell	Kontkanen et al., 2002
***Nur*** **family**	Acute **Typical antipsychotics** on Nurr1, Nur77 and Nor-1	↑	Striatum	Maheux et al., 2005
	Acute **Clozapine** on Nor1 and Nurr77	↑	NAc Shell	Werme et al., 2000
	Acute **Haloperidol** On Nor1 and Nurr77	↑	NAc Shell and Core, medial, and lateral Caudate Putamen.	Werme et al., 2000
	Acute **Clozapine** or **Haloperidol** on Nurr77	↑	PFC, cingulate cortex and NAc Shell	Beaudry et al., 2000
	Acute and chronic **Haloperidol** on Nurr77	↑	Lateral striatum	
	Chronic **Haloperidol** or **Clozapine** on Nurr77	↓	Primary Somato-sensory cortex	Langlois et al., 2001
***Egr1***	Acute **Haloperidol** or **Clozapine**	↑	Striatum, NAc	Nguyen et al., 1992; MacGibbon et al., 1994
	Acute **Haloperidol, Asenapine or Olanzapine**	↑	Striatum, NAc Shell	De Bartolomeis et al., 2015a
	Acute **Lurasidone**	↑	Striatum	Luoni et al., 2014b
	Chronic **Haloperidol**	↑	PFC	Verma et al., 2007
	Chronic low dose **Lurasidone**	↑	PFC, Striatum	Luoni et al., 2014b
	Chronic **Olanzapine**	↓	PFC, Locus Coeruleus	Verma et al., 2006, 2007
***Arc***	Acute **Asenapine**	↓	PFC	De Bartolomeis et al., 2015a
	Acute high dose **Asenapine**	↑	Striatum, NAc Core	De Bartolomeis et al., 2015a
	Acute **Clozapine**	↓	Thalamus, mPFC, Cingulate cortex	Robbins et al., 2008
	Acute **Haloperidol** or **Olanzapine** or High dose **Amisulpiride**	↑	Striatum	Robbins et al., 2008; Fumagalli et al., 2009; Iasevoli et al., 2010b; De Bartolomeis et al., 2013b
	Acute **Haloperidol**	↑	Striatum, NAc Core and Shell	Polese et al., 2002; Dell'aversano et al., 2009; Iasevoli et al., 2010a,b, 2011; De Bartolomeis et al., 2015a
	Acute **Haloperidol** or **Olanzapine**	↓	PFC	Fumagalli et al., 2009
	Chronic **Haloperidol** or **Olanzapine**	↓	Striatum	Fumagalli et al., 2009
	Acute **Lurasidone**	↑	Hippocampus, Striatum	Luoni et al., 2014b
	Chronic **Aripiprazole**	↑	PFC, Striatum, Hippocampus	Luoni et al., 2014a
	Chronic **Asenapine or Olanzapine or Haloperidol**	↓	PFC	Buonaguro et al., 2017a
	Chronic **Lurasidone**	↑	PFC, Hippocampus, and Striatum	Luoni et al., 2014b
***Homer1a***	Acute **Asenapine or Olanzapine**	↑	PFC, Lateral Striatum, NAc	Iasevoli et al., 2010a; De Bartolomeis et al., 2015a
	Acute **Clozapine**	↑	NA	Polese et al., 2002
	Acute **Haloperidol**, but not **Clozapine**,	↑	Lateral Striatum	Cochran et al., 2002
	Acute **Risperidone**	↑	Lateral Striatum, NAc	Iasevoli et al., 2010a
	Acute **Sertindole**	↑	PFC	Iasevoli et al., 2010b
	Acute **Ziprasidone**	↑	Striatum	Iasevoli et al., 2011
	Sub-chronic **Amisulpiride**	↑	PFC, Striatum	De Bartolomeis et al., 2016
	Chronic **Haloperidol**	↑	Striatum	Iasevoli et al., 2010b; Buonaguro et al., 2017a
	Chronic **Olanzapine** or high dose **Asenapine**	↑	Striatum	Buonaguro et al., 2017a
***BDNF***	Acute **Haloperidol**	↓	Thalamus	Robbins et al., 2008
	Acute or chronic **Clozapine**	*nc*	Cortex, Hippocsmpus	Linden et al., 2000
	Acute and chronic **Clozapine** or **Haloperidol**	↓	Hippocampus	Lipska et al., 2001
	Chronic **Aripiprazole**	↓	Hippocampus	Luoni et al., 2014a
	Chronic **Clozapine**	↑	Whole rat brain	Kim et al., 2012; Rizig et al., 2012
	Chronic **Haloperidol** or high-dose **Risperidone**	↓	Hippocampus	Chlan-Fourney et al., 2002; Parikh et al., 2004
	Chronic **Lurasidone**	↑	PFC, Hippocampus	Fumagalli et al., 2012
	Chronic **Olanzapine** or **Clozapine**	↑	Hippocampus	Bai et al., 2003
	Chronic **Quetiapine**	↑	Hippocampus	Park et al., 2006
***Npas4***	Acute **Lurasidone**	↓	Hippocampus	Luoni et al., 2014b
	Chronic **Aripiprazole**	↑	Dorsal Hippocampus	Luoni et al., 2014a
***Narp***	Acute **Clozapine**	↓	Striatum	Robbins et al., 2008
	Acute **Haloperidol**	↓	Thalamus	

*↑, Gene expression is up-regulated; ↓, Gene expression is down-regulated; NAc, Nucleus accumbens; PFC, Prefrontal cortex, FC Frontal cortex*.

### *C-fos:* the prototypical IEG

#### C-*fos* regulation by dopamine

*C-fos* is a proto-oncogene encoding for a TF that is induced in response to multiple stimuli, included neural activity (Durchdewald et al., 2009). In resting conditions, the product of the *c-fos* gene, the Fos protein, is expressed in small amounts in the brain.

*C-fos* transcription may be activated in response to many different extracellular signals, including growth factors and neurotransmitters, such as dopamine. Several early studies demonstrated that transcriptional regulation after dopaminergic stimuli, such as amphetamine/cocaine administration, is a pivotal mechanism by which neurons may respond to environmental adaptations (De Bartolomeis et al., 2013b). The phosphorylation of the cAMP response element binding protein (CREB) is crucial to couple dopamine stimulation to the IEG transcription. Indeed, signals starting at dopamine receptors may promote CREB phosphorylation, which in turn regulates *c-fos* transcription. When translated, the Fos protein may dimerize with members of Jun family in order to start the formation of the Activator Protein-1 heterocomplex (AP-1), which in turn may trigger the expression of genes involved in cell proliferation and differentiation, as well as in activity-stimulated synaptic rearrangements (Herrera et al., 1990).

Based on its fast induction dynamics, *c-fos* expression has been widely used to characterize the different topographic patterns of neural activation following treatments with different antipsychotics (Nguyen et al., 1992; Merchant et al., 1994).

#### C-*fos* in schizophrenia and its modulation by antipsychotics

Recent human studies have pointed out that specific polymorphisms of *c-fos* gene may be either negatively or positively associated to schizophrenia, since decreased Fos protein blood levels may be found in schizophrenia patients (Boyajyan et al., 2015), thus reinforcing the possibility of an implication of this IEG in schizophrenia pathophysiology and, possibly, in its treatment.

Early studies observed that typical and atypical antipsychotics may induce different patterns of *c-fos* activation in cortical and subcortical brain regions. Indeed, typical antipsychotics, such as haloperidol, may induce *c-fos* expression in the dorso-lateral regions of the striatum, as well as in the nucleus accumbens and in the lateral septum. Atypical antipsychotics, such as clozapine, were found to induce *c-fos* expression in prefrontal cortex and medial striatum (Robertson et al., 1994). Since the dorso-lateral striatum has been implicated in motor control (Balleine and O'doherty, 2010), it has been suggested that the liability of an antipsychotic drug to induce extrapyramidal side-effects (EPSEs) might be predicted by its propensity to induce *c-fos* expression in the motor circuits of the striatum (Robertson and Fibiger, 1996). On the other hand, the induction of *c-fos* expression in prefrontal cortex and limbic striatum by atypical antipsychotics (e.g., clozapine; Robertson et al., 1994) has been potentially correlated with the ability of these compounds to impact, at least in part, brain circuitry implicated in the pathophysiology of negative symptoms of schizophrenia, based on the hypothesis explaining negative symptoms with a potential hypo-frontality in schizophrenia patients (Weinberger and Berman, 1996). However, this observation should nowadays be discussed with caution, considering recent advances in molecular characterization of old and novel antipsychotics, as well as the latest results on the real effect size of atypical antipsychotics on negative symptoms (Kantrowitz, 2017).

Typical and atypical antipsychotics differentially enhance *c-fos* expression in the two histological compartments of the striatum, striosome, and matrix. Indeed, typical antipsychotics induce *c-fos* at a similar extent in the striosome and in the matrix, while most atypical antipsychotics preferentially induce *c-fos* in the striosome (Hiroi and Graybiel, 1996; Bubser and Deutch, 2002). It is noteworthy that no difference in the striosome/matrix ratio (SMR) has been found for typical antipsychotics between dorso-lateral caudate-putamen and dorso-medial caudate-putamen, while clozapine showed a significantly higher SMR in dorso-lateral than in the dorso-medial region of the caudate-putamen (Bubser and Deutch, 2002). Numerous studies have correlated the matrix with motor behavior and stimulus-response memory consolidation, while the striosome has been related to reward mechanisms (White and Hiroi, 1998). Thus, the striosome/matrix architecture of the striatum has been proposed as a morphological substrate for a modular reinforcement-learning model (Amemori et al., 2011). Moreover, it has been suggested that the striosome may be linked to cognition, since it receives prominent inputs from association cortex (Bubser and Deutch, 2002).

Differences in the putative clinical profile of typical and atypical antipsychotics may be inferred by their specific spatial pattern of *c-fos* induction.

D2Rs blockade by antipsychotics has been demonstrated to relieve the inhibition of adenylyl cyclase and activate the PKA, which in turn is responsible for the phosphorylation of CREB. Phospho-CREB may then interact with the cAMP response element (CRE) site in the promoter region the *c-fos* gene (Benito and Barco, 2015). Considering that all the antipsychotics, with few exceptions, are multireceptor-binding drugs, it is conceivable that other receptors beyond D2Rs could be responsible and/or contribute to *c-fos* activation.

Nevertheless, mapping *c-fos* expression to unravel antipsychotics differential functional impact on brain areas has some limitations. In fact, although antipsychotics show specific topographical patterns of *c-fos* induction according to their typical/atypical characteristics, the expression of *c-fos* in neurons has been described to be coupled to multiple different extracellular stimuli, hence it is difficult to attribute a specific *c-fos* “fingerprint profile” to each antipsychotic compound. *C-fos* induction may help to detect where and when a brain area is activated by a certain compound, but it gives little information on which is the specific intracellular pathway stimulated by this compound. Thus, the data obtained by *c-fos* induction in response to antipsychotics need to be integrated with by other IEGs induction with a more direct function in the synapse.

### Δ*FosB:* an IEG with a dual function

Δ*FosB* is a splicing variant of the *FosB* gene, a member of the FRA family (Fos Related Antigens). Depending on its expression kinetics, the role of ΔFosB can be either of transcription activator or repressor, with lower levels leading to short-term gene repression and higher levels leading to long-term gene activation (Nestler, 2015).

Among the main target genes of Δ*FosB* there are: *metabotropic Glutamate Receptors subtype 2 (mGluR2), Dynorphin*, the *nuclear factor kB (NFkB)* and *c-fos*.

Δ*FosB* gene is rapidly induced in the dorsal striatum and nucleus accumbens dynorphin-expressing Medium-sized Spiny Neurons (MSNs)—two neural populations closely involved into reward and addiction—in response to addictive drugs such as cocaine and amphetamines (Maze and Russo, 2010). Δ*FosB* induction is reported to not undergo tolerance, thus its accumulation in those brain regions is quite stable after repeated administrations. Moreover, unlike other IEGs, Δ*FosB* levels in striatum and nucleus accumbens are quite stable across the time, and remain up-regulated for weeks after the initial stimulus (Nestler, 2005).

#### Δ*FosB* modulation by antipsychotics

Δ*FosB* expression has been also studied in response to antipsychotic drugs. Indeed, chronic treatment with haloperidol may enhance the expression of Δ*FosB* in the ventral, medial, and dorso-lateral aspects of the striatum (Rodriguez et al., 2001). Clozapine, on the other hand, may induce ΔFosB-like immunoreactivity not only in the ventral striatum but also in the prefrontal cortex and lateral septum, with a weaker impact on dorso-lateral striatum, whereas risperidone and olanzapine only weakly induce Δ*FosB* in striatum (Vahid-Ansari et al., 1996; Atkins et al., 1999). Given the long-term standing of Δ*FosB* activation in response to dopaminergic drugs, this IEG has been proposed as a specific marker to identify neurons undergoing prolonged activation in chronic paradigms (Dietz et al., 2014).

### C-Jun

#### *C-Jun* regulation and schizophrenia preclinical modeling

C-Jun is a TF that dimerizes with Fos family members to form the AP-1 complex. As an IEG, c*-Jun* plays a pivotal role in neuronal apoptosis and neurons survival (Jochum et al., 2001). Various extracellular stimuli may activate the JNK (c-jun kinase)/C-Jun cascade, including stress, ischemia, and stroke, seizures, learning and memory, axonal injury (Raivich and Behrens, 2006). Interestingly, a potential indirect role for *c-Jun* in a preclinical model of schizophrenia pathophysiology has been recently highlighted by investigating attentive function in mice haploinsufficient for *Map2k7* (*Map2k7*^+/−^ mice). Specifically, *Map2k7* encodes for MKK7 (MAP kinase kinase 7), which is responsible for the activation of JNK. The reduction of *Map2k7* function has been found to be associated to cognitive deficits in mice (Openshaw et al., 2017).

Moreover, putative links between *c-Jun* and schizophrenia are suggested in preclinical models by several findings showing that both psychotomimetic and antipsychotic drugs modulate *c-Jun* levels in brain regions implicated in schizophrenia. Indeed, *c-Jun* expression is affected by N-methyl-D-aspartate (NMDA) receptor antagonists such as MK-801 (Gerlach et al., 2002) mimicking a preclinical model of schizophrenia.

#### *C-Jun* modulation by antipsychotics

*C-Jun* expression has been demonstrated to be modulated by antipsychotics. Chronic treatment with clozapine or haloperidol induces long-lasting *c-Jun* expression in the rat forebrain and basal ganglia even after a washout period (Kontkanen et al., 2002). A recent proteomic quantification analysis has demonstrated that chronic haloperidol administration in rodents may modulate the expression of 216 proteins in hippocampus, including c-Jun N-terminal kinase signaling (Schubert et al., 2016). In contrast, acute haloperidol or clozapine treatment have been shown to exert no effects on *c-Jun* expression, although both these treatments produce clear changes in the expression of several other IEGs, including *c-fos* and other Jun-family members (MacGibbon et al., 1994). These data suggest that antipsychotic drugs may play different roles in modulating apoptosis-related molecules, and are coherent with the findings suggesting that typical and atypical antipsychotics may differentially affect putative neuroprotection (Jarskog, 2006; Nandra and Agius, 2012).

Thus, taken together, the findings reviewed until now may suggest that:

1. *c-fos* activation could represent a valuable tool to understand how antipsychotics recruit different brain regions;
2. *c-fos* activation mirrors, with acceptable approximation, the involvement by antipsychotics of motor vs. limbic brain regions based on the different receptor profile of the antipsychotic taken into account;
3. Based on the emerging role of *c-fos* polymorphisms in schizophrenia, it will be of interest to investigate whether and how the association with *c-fos* and related genes may have any causative role in the pathophysiology of the disorder;
4. Δ*FosB* expression is probably of higher interest to explore the long-term effects of antipsychotics action;
5. Despite being less investigated compared to other IEGs, *c-Jun* stands by itself for the recent proteome findings linking its signaling pathway to antipsychotics action in a network fashion.

## Nuclear receptors (Nur) superfamily: antipsychotics modulation of dopaminergic neurodevelopmental factors

### The Nur superfamily: role in dopamine system development and modulation by antipsychotics

Nurr1, NGFI-B/Nur77 (Nerve Growth Factor Inducible-B) and Nor1 (neuron-derived orphan receptor-1) are members of the NR4A (Nuclear Receptors 4A) subgroup of nuclear orphan receptors superfamily, which includes a wide variety of TFs, such as retinoid hormone receptor, steroid, and thyroid hormone receptor (Law et al., 1992; Maxwell and Muscat, 2006). All the three Nur members share overlapping sequences and play essential roles in the development of the dopaminergic system. The three members of NR4A subgroup have been described to respond to several physiological and physical stimuli, such as prostaglandins, stress, hormones, neurotransmitters, membrane depolarization, and magnetic fields (Katagiri et al., 1997; Tetradis et al., 2001; Kagaya et al., 2005) in an IEG-like fashion. Thus, the immediate-early response of *NR4A* genes to environmental stimuli is an essential feature of these nuclear receptors, which has been extensively studied with regard to their correlation with dopaminergic system (Campos-Melo et al., 2013).

*Nurr1* is mainly expressed in the central nervous system, especially in midbrain dopaminergic neurons of the substantia nigra and the ventral-tegmental area (VTA; Backman et al., 1999). Several studies have reported that *Nurr1* plays an essential role in the development and differentiation of dopaminergic neurons of the midbrain. Given its role in dopamine neurons development, *Nurr1* has been implicated in neuropsychiatric disorders in which dopamine system is dysfunctional, such as schizophrenia. Recent studies reported that *Nurr1* gene may be considered as a possible candidate to explore the dysfunctional gene-environment interaction that is considered to be at the basis of these disorders. Indeed, two missense mutations in the gene have been found in schizophrenia patients (Buervenich et al., 2000) and may be directly related to their impaired cognitive performances (Ancin et al., 2013).

Heterozygous deletion of *Nurr1* gene in mice has been recently considered as a possible animal model of schizophrenia, since these animal display elevated dopamine levels in basal ganglia (Moore et al., 2008), and characteristic dysfunctional behaviors resembling psychotic symptoms in humans (Rojas et al., 2007).

Similarly, also N*ur77* has been implicated in the pathophysiology of schizophrenia. Indeed, reduced levels of *Nur77* have been detected in prefrontal cortex of post-mortem schizophrenia patients (Xing et al., 2006). Moreover, single nucleotide polymorphisms of *Nur77* gene have been associated with elevated risk of tardive dyskinesia (TD) in schizophrenia patients (Novak et al., 2010).

Given the direct correlation between *Nur* IEGs and the development of dopaminergic system, a large body of evidence has been set up on the regulation of *NR4A* IEGs in response to dopamine manipulation.

### *Nur* IEGs modulation by antipsychotics

The first studies with antipsychotics demonstrated that *Nur* IEGs response to these drugs may resemble that of *c-fos* in rat brain. For instance, typical and atypical antipsychotics induce differential patterns of *Nur* IEGs expression. Indeed, acute haloperidol administration pronouncedly increases *Nur77* expression in dorso-lateral striatum, whereas clozapine induces this gene preferentially in prefrontal cortex and in the shell of the nucleus accumbens (Beaudry et al., 2000). Moreover, haloperidol selectively increases *Nur77* dorsolateral striatal expression in enkephalin-containing neurons, which are MSN neurons mostly expressing D2Rs, whose up-regulation has been correlated to extrapyramidal symptoms induced by neuroleptics. In addition, the same report showed that chronic haloperidol administration provokes a further increase in *Nur77* dorso-lateral striatal expression, whereas chronic clozapine reduces *Nur77* gene expression below basal values in prefrontal and accumbal areas. To confirm the role of *Nur77* in acute neuroleptic-induced EPSEs, later studies demonstrated that in *Nur77*-deficient mice haloperidol-induced acute catalepsy was completely abolished, as well as the *Nur77* mRNA overexpression in enkephalin-positive neurons (Ethier et al., 2004). Therefore, similarly to *c-fos* expression, *Nur* IEGs modulation by antipsychotics may be used as a tool to dissect the propensity of a neuroleptic drug to induce extrapyramidal side effects.

Some other significant similarities with *c-fos* have been shown in the regulation of *NR4A* members expression by antipsychotics. Indeed, Maheux and coworkers demonstrated that *Nur* IEGs may be induced by typical antipsychotics selectively in striatal areas that control motor functions, whereas atypical antipsychotics induced *Nur* IEGs expression in limbic areas (Maheux et al., 2005). This induction pattern tightly correlates with D2Rs affinity by each antipsychotic in striatum and with D2/D3Rs affinity in the nucleus accumbens. The same research group further demonstrated that selective serotonergic and adrenergic drugs may modulate haloperidol-induced *Nur* IEGs expression, suggesting that also serotonin neurotransmission may take part into the differential patterns of regulation of these genes by typical and atypical antipsychotics (Maheux et al., 2012).

There are substantial differences in response to antipsychotics between the different members of *Nur* family: both *Nur77* and *Nor1* are *de novo* induced in dopamine neurons and striatal areas, whereas *Nurr1* is basally expressed in VTA and substantia nigra and its expression is enhanced by antipsychotics (Eells et al., 2012).

Regarding the mechanisms involved in *Nur* regulation by antipsychotics drugs and D2Rs antagonists, it has been demonstrated that, at least for *Nur-77* and *Nor-1*, the induction/increase in mRNA are depending by both mitogen-associated and extracellular signal-regulated kinases (MEK) and Protein Kinase C (PKC) in the case of *Nurr-77* and by PKC only in the case of *Nor-1* (Bourhis et al., 2008).

Hence, *Nur* IEGs modulation by antipsychotics appears to provide a complementary information as compared to *c-fos* expression patterns, thereby contributing to shed further light on the impact of these drugs not only on brain areas that are targeted by dopamine neurons, but also on areas in which dopamine neurons localize. Thus, although still elusive, the analysis of *Nur* IEGs modulation by antipsychotic drugs may be a further tool to dissect the mechanisms of action of these compounds on dopamine systems, as well as it may help to further clarify the molecular mechanisms by which these drugs alter locomotor activity in animal models and in humans.

## Modulation by antipsychotics of IEGs involved in synaptic plasticity: putative targets for cognitive deficits in psychosis

Abnormal synaptic plasticity may account for several cognitive and behavioral processes that are dysfunctional in schizophrenia. Cognitive impairment, indeed, is a striking clinical aspect of psychotic illnesses, is detectable before the onset of other symptoms, and it is considered among the best predictors of long-term lifetime functioning (Green, 1996).

Since long-term neural plasticity requires protein synthesis, IEGs expression could be considered as a necessary step in synaptic architecture remodeling. For several IEGs involved in these processes, a role in the pathophysiology of psychosis has been proposed. In the following section, we will summarize how antipsychotic therapy may impact these genes expression.

### *Egr1*: how antipsychotics impact synaptic processes underpinning memory and learning

#### *Egr1* regulation and involvement in synaptic plasticity

The Egr-family consists of four highly homologous zinc-finger TFs: *Egr1* (Early growth response gene 1, also named NGFI-A, zif-268, Krox 24), *Egr2* (Krox 20), *Egr3* (PILOT), and *Egr4* (NGFI-C).

*Egr1* is an immediate-early gene (IEG) coding for a TF and is constitutively expressed in the cortex, amygdala, striatum, nucleus accumbens, hippocampus, and cerebellum (Beckmann and Wilce, 1997). Several stimuli have been demonstrated to induce *Egr1* overexpression in these areas, such as seizures, ischemia, stress, and drug administration (Hughes and Dragunow, 1995). Many of these stimuli share the common feature of elevating intracellular calcium (Ca2+; Ghosh et al., 1994). Pharmacological stimuli provoking massive Ca2+ influx in neurons have been described to increase *Egr1* expression (Shirayama et al., 1999; Zhou et al., 2009; Gangarossa et al., 2011). *Egr1* activity has been related to the transcription of other IEGs involved in synaptic plasticity, above of all *Arc* (Penke et al., 2011). In turn, *Egr1* expression may be under the control of other genes, such as the *Brain-Derived Neurotrophic Factor* (*BDNF*; Robinet and Pellerin, 2011) and other proteins involved in intracellular signaling (Lam et al., 2009).

*Egr1* synaptic action has been related to neural plasticity, in particular to synaptic processes leading to memory consolidation and behavioral adaptations (Davis et al., 2003; Okada et al., 2015) Intriguingly, dysfunctions in the postsynaptic machinery deputed to control memory and learning processes have been recently related to cognitive impairment in major neuropsychiatric disorders, such as schizophrenia (Grant, 2012). In paradigms of instrumental learning, *Egr1* is markedly induced in frontal and cingulate cortices (Hernandez et al., 2006; Snyder et al., 2012). Furthermore, *Egr1* plays a pivotal role in maintaining the late phase of LTP in hippocampus, in dorsal caudate-putamen (Gill et al., 2007), and in the retrosplenial cortex (Amin et al., 2006). *Egr1*, along with *BDNF*, also appears to play a role in retrieval-dependent plasticity, a mechanism accounting for the modification of previously consolidated memories being recalled (Lee, 2010). Moreover, it has been shown that *Egr1*, together with *BDNF* (Barnes et al., 2012), *Homer1a* and *Arc*, has a relevant role in the mechanisms of the initial consolidation, reconsolidation and extinction of fear- and anxiety-related memory (Lonergan et al., 2010; Maddox et al., 2011; Cheval et al., 2012).

Dopamine and glutamate systems have been implicated in synaptic processes involved in memory consolidation [e.g., long-term Potentiation (LTP) and long-term depression (LTD)], and several studies reported that *Egr1* expression may be modulated by stimuli affecting either glutamate or dopamine neurotransmission (Li et al., 2016).

#### *Egr1* modulation by antipsychotics

Several antipsychotics (e.g., clozapine) modulate synaptic proteins related to memory formation in hippocampus and improve cognitive tasks in animal models of pharmacological NMDA receptor hypofunction (Ozdemir et al., 2012). With regard to dopamine, *Egr1* gene and protein expression are modulated by both acute and chronic antipsychotic treatments in preclinical settings (Wheeler et al., 2014; De Bartolomeis et al., 2015a).

Clinical studies have reported an abnormal regulation of *Egr1* in schizophrenia patients compared to normal controls. Specifically, post-mortem gene expression studies and *in vivo* plasma detection have demonstrated that *Egr1* is down-regulated in prefrontal cortex of schizophrenia patients in a fashion that is directly correlated with decrease in GAD1 (glutamate decarboxylase 1, the enzyme that is responsible for GABA production), and plasma levels were reduced, therefore supporting the view that *Egr1* may be a potential biomarker of the disease (Kimoto et al., 2014). Interestingly, an association of SNPs in *Egr3* and *Arc* with schizophrenia has been proposed as a biological pathway of environmentally responsive, synaptic plasticity-related, schizophrenia risk genes (Huentelman et al., 2015).

Given the potential role of this IEG in schizophrenia pathophysiology and treatment, it is to mention that early studies have also compared *Egr1* modulation by antipsychotics to the modulation of the other well-known IEG *c-fos*, showing that these two IEGs, although similarly impacted by typical and atypical antipsychotics in cortex and striatum, displayed some substantial differences. Haloperidol has been reported to induce both *c-fos* and *Egr1* expression in striatum, whereas clozapine may induce *Egr1* but not c-*fos* expression in the same region. Both antipsychotics may induce the expression of both these genes in nucleus accumbens (Nguyen et al., 1992; MacGibbon et al., 1994). Subsequent findings further demonstrated a differential response of *Egr1* from *c-fos* also in chronic antipsychotics administration paradigms. *Egr1*, indeed, is robustly down-regulated in locus coeruleus and prefrontal cortex of olanzapine chronically administered rats, whereas *c*-*fos* expression remains up-regulated (Verma et al., 2007). Moreover, chronic haloperidol may increase cortical *Egr1* expression, whereas it decreases *c*-*fos* expression in this area (Verma et al., 2007).

Targeted experiments have demonstrated that *Egr1* modulation by antipsychotic drugs may be directly related to the synaptic functions of drug-associated memory consolidation. For instance, high D2R-blocking antipsychotics (e.g., sulpiride) may prevent the increase in *Egr1* expression induced by acute cocaine administration in striatum, but not in the cortex (Daunais and McGinty, 1996). The modulation of *Egr1* and other activity-regulated genes such as *Arc* and *Npas4* has been studied in rodents after the administration of the novel antipsychotic lurasidone, which is characterized by a multi-receptor profile and particularly by a potent 5-HT7 receptor antagonism, considered beneficial for mood and cognition (Luoni et al., 2014b).

Finally, *Egr1* levels of expression have been recently investigated in preclinical settings exploring new therapeutic strategies in schizophrenia beyond current antipsychotic drugs (Gentzel et al., 2015).

However, it should be considered that the dopaminergic regulation of *Erg1* is even more complicated by the action of multiple pathways in reciprocal interplay with dopaminergic system. Recent evidence, indeed, demonstrated that *mu*-opioid receptors have also a major implication in psychostimulant-induced sensitization (Shen et al., 2010) and antipsychotic drugs seem unable to prevent methamphetamine-induced striatal overexpression of *Egr1* in *mu*-opioid receptors knock-out animals (Tien et al., 2010).

Putting together the findings reviewed therein, the role of *Egr1* in antipsychotics action appears relevant mainly because it is directly implicated in specific signaling and neuronal plasticity programs related to memory, cognition and executive-like functions in preclinical models. This is a major point, since there is a need to explore new compounds in schizophrenia that may address dysfunctions in the domains of cognitive and negative symptoms, which are poorly affected by currently available antipsychotic agents.

### *Arc/Arg3.1*: modulation of long-term activity-dependent synaptic efficacy by antipsychotic treatments

#### *Arc* regulation and involvement in synaptic plasticity: relevance for schizophrenia

As other IEGs, *Arc* (activity-regulated cytoskeletal-associated protein) also referred to as *Arg3.1* (activity-regulated gene homolog 3.1), is expressed at low levels in neurons, especially in the hippocampus. However, *Arc* levels are relatively higher in cortex, and are directly linked to NMDA receptor activation (Link et al., 1995). *Arc* shows unique features, since its mRNA may be induced together with other IEGs by neural activation (i.e., single seizures), but differently from other mRNAs—such as *Egr1*—that remain in the neuron soma, *Arc* is rapidly translocated to dendritic spines (Wallace et al., 1998). The activity-dependent translocation of *Arc* requires NMDA receptor activation (Steward and Worley, 2001). Moreover, Arc protein is selectively produced in dendritic spines near the activation site, even in the presence of protein-synthesis inhibitors, thereby indicating that *Arc* mRNA owns a unique intrinsic signal that permits the activity-related targeting to stimulated dendrites (Bramham et al., 2010; Steward et al., 2014). The activity-dependent regulation of *Arc* expression has been extensively studied, thus leading to several findings reporting its involvement in synaptic plasticity and its implication in memory and learning processes, which have been demonstrated to be altered in schizophrenia. Experience-related stimuli may potently increase *Arc* expression in brain areas involved in memory consolidation (Lyford et al., 1995). Indeed, the exploration of new environments strongly induces *Arc* expression in hippocampus and cortex (Vazdarjanova et al., 2002). Consistently, *Arc* knock-out animals fail to form long-term memories for learning tasks, displaying impaired LTP and LTD, but unaltered short-term memory (Plath et al., 2006). A role for *Arc* in synaptic scaling, a form of homeostatic synaptic plasticity, has been postulated (Gao et al., 2010).

Hence, *Arc* functions seem to be required for synaptic plasticity processes starting at multiple neurotransmitter receptors, such as alpha-amino-3-hydroxy-5-methyl-4-isoxazolepropionic acid (AMPA), NMDA, dopamine, serotonin, acetylcholine, and adrenaline receptors (Chowdhury et al., 2006; Rial Verde et al., 2006).

Dopamine and glutamate are considered among the principal neurotransmitters implicated in memory and learning processes, as well as in schizophrenia pathophysiology in which aberrant salience has been reported. Dopamine has been demonstrated to be pivotal for working memory in rodents and in non-human primates (Castner et al., 2004; Rinaldi et al., 2007). *Arc* response to memory and learning stimuli may account for a direct involvement of this IEG into dopaminergic-dependent mechanisms of memory consolidation, as well as for its implication in dopamine-glutamate subcellular interactions that control synaptic plasticity processes thought to be dysfunctional in the pathophysiology of schizophrenia (Eastwood, 2004; Grant, 2012; Yin et al., 2012). To confirm the role of *Arc* in synaptic processes of dopamine-dependent memory formation, it has been demonstrated that amphetamines may modulate *Arc* learning-induced expression in hippocampus (Wiig et al., 2009). Bloomer and coworkers have reported a combined effect of NMDA receptors and D1Rs on *Arc* expression in hippocampal neurons (Bloomer et al., 2008), as well as a dramatic reduction in *Arc* expression by dopamine agonists when NMDA receptor blockers are concurrently administrated, thus confirming the role of dopamine-glutamate correct interaction in memory consolidation and the crucial functions of *Arc* in this process. *Arc* gene has been reported to be affected by *de novo* mutations in schizophrenia patients and it is part of an enriched gene set characterized by rare disruptive mutations contributing to the genetic risk for schizophrenia (Fromer et al., 2014; Purcell et al., 2014). Further evidence of an implication of *Arc* in schizophrenia pathophysiology comes from preclinical studies (Manago and Papaleo, 2017). Disruption of *Arc* produces deficits in sensorimotor gating, cognitive functions, social behaviors, and amphetamine-induced psychomotor responses in mice that are reminiscent of some features of psychosis (Manago et al., 2016).

#### *Arc* modulation by antipsychotics

*Arc* expression in response to antipsychotic challenges has been extensively investigated, in order to shed further light on the molecular mechanisms involved in antipsychotic-mediated modulation of the synaptic plasticity processes putatively disrupted in schizophrenia.

Early works demonstrated that Phencyclidine (PCP)-induced *Arc* overexpression in prefrontal cortex and nucleus accumbens may be inhibited by pretreatment with clozapine, olanzapine, and risperidone, but not by haloperidol (Nakahara et al., 2000), thus suggesting that *Arc* modulation may be useful to dissect typical from atypical antipsychotics impact on psychotomimetic drug-induced synaptic dysfunctions. Subsequently, acute administration of both typical and atypical antipsychotics was demonstrated to induce *Arc* gene expression in striatum, with haloperidol showing a more prolonged effect on *Arc* induction than olanzapine (Fumagalli et al., 2009). These results have been directly correlated to the degree of D2R blockade induced by each antipsychotic, since a selective D2Rs antagonist, such as raclopride, may induce *Arc* striatal expression and reduce its cortical expression, whereas a selective D2Rs agonist, such as quinpirole, may reduce *Arc* striatal expression and has no effects on its cortical expression. Notably, Fumagalli et al. (2009) further demonstrated that a prolonged treatment with haloperidol and olanzapine markedly reduced *Arc* striatal expression, as well as only olanzapine may reduce the expression of the gene in the cortex.

With regard to cortical and subcortical expression of *Arc*, work from our laboratory demonstrated that *Arc* gene expression may be induced by haloperidol, but not by sertindole in the striatum, thus further suggesting that *Arc* modulation may be tightly related to the tuning of dopamine neurotransmission exerted by each antipsychotic (Iasevoli et al., 2010b). Sertindole shows a milder D2Rs impact than haloperidol in the striatum, with a quite absent blockade of D2 autoreceptors (Valenti and Grace, 2010). The involvement of serotonin 5-HT2A receptors has been demonstrated in *Arc* cortical modulation, with NMDA receptor function being relevant in these effects, thereby suggesting a crucial role of *Arc* in synaptic rearrangements induced by combined serotonin-dopamine-glutamate stimuli in the cortex by antipsychotics (Pei et al., 2004).

*Arc* has been shown to be responsive to acute (with significant increase of the transcript in striatum) and chronic (with prevalent gene expression increases in prefrontal cortex and hippocampus and decreases in striatum) lurasidone treatment, suggesting a region-specific fingerprint of *Arc* induction (Luoni et al., 2014b). An intra-striatal specificity of *Arc* activation was detected after acute administration of amisulpride (35 mg/kg) with prevalent increase of the transcript in the medial caudate-putamen compared to more pronounced induction in dorsal caudate-putamen by haloperidol (0.8 mg/kg; De Bartolomeis et al., 2013b). Again, a region-specific induction of Arc protein was detected in the shell of the nucleus accumbens by clozapine (20 mg/kg) compared to haloperidol (1 mg/kg; Collins et al., 2014). Finally, *Arc* has been instrumental also for exploring the brain region effect of innovative treatment approaches in schizophrenia, such as augmentation strategies to antipsychotics (i.e., minocycline in combination with haloperidol; Buonaguro et al., 2017b).

Direct evidence exist that *Arc* induction can be responsible for increasing the density and for reducing the width of dendritic spine possibly by a mechanism involving AMPA endocytosis (Peebles et al., 2010).

These data globally suggest that *Arc* is involved in the neural plasticity mechanisms induced by antipsychotics in distinct brain regions. *Arc* modulation may be demonstrated to occur before the timing necessary to observe the therapeutic-like effects commonly observed during antipsychotic therapies, possibly suggesting that *Arc* is potentially relevant in establishing the correct synaptic rearrangements underlying antipsychotic effects. Finally, the recent discovery of *Arc* subdomains similar to the domain of HIV capsid and its involvement in rapid synaptic functions (possibly derived from the ancestral viral origin) deranged in schizophrenia (Zhang et al., 2015), make this IEG of relevant interest for studying specifically “fast” synaptic changes during antipsychotic treatment.

### *Homer1a*: IEG-mediated activity-dependent postsynaptic and architecture rearrangements in response to antipsychotic treatment

#### The *Homer* family and its regulation by dopamine-glutamate interaction

*Homer* genes encode a family of scaffolding proteins (Homer1, Homer2, Homer3) localized mainly at the glutamatergic postsynaptic density (PSD) of dendritic spines, where they act as multifunctional adaptors among multiple transduction pathways. *Homer1* gene encodes both constitutively expressed long transcripts (*Homer1b/c*) and for a short isoform named *Homer1a*, which is induced in an IEG-like fashion (Bottai et al., 2002). Within the PSD, Homer proteins couple to metabotropic and indirectly ionotropic glutamatergic receptors, bridging both to intracellular receptors, such as the inositol 1,4,5-trisphosphate receptor (IP3Rs), the ryanodine receptor (RyR), and to other PSD scaffolding proteins, such as Shank (De Bartolomeis and Iasevoli, 2003; Gao et al., 2013). When induced, Homer1a protein disassembles constitutive Homers clusters by acting as a “dominant negative,” thus modifying synaptic architecture and Ca2+ homeostasis (Shiraishi-Yamaguchi and Furuichi, 2007). Several studies demonstrated a pivotal role of *Homer1a* in modulating the crosstalk between PSD proteins involved in mechanisms underlying synaptic plasticity, such as receptor localization, distribution and internalization (Iasevoli et al., 2013). For example, it has been observed that *Homer1a* is rapidly up-regulated during enhancement of network activity and promotes the agonist-independent signaling of group I mGluRs that may in turn scale down the expression of AMPA receptors (Hu et al., 2010). To note, in animal models, impaired homeostatic scaling has been reported in a NMDA receptor-blocking experimental paradigm, which mimics psychotic states (Wang and Gao, 2012). Based on the crucial role of *Homer1a* in synaptic plasticity, dysfunctions in its fine-tuning activity have been closely related to psychiatric disorders (Luo et al., 2012). *Homer1* polymorphisms have been associated with schizophrenia (Spellmann et al., 2011) and cocaine addiction (Dahl et al., 2005) and *Homer1* knock-out mice exhibit a behavioral phenotype resembling psychotic disorders (Szumlinski et al., 2005), as well as Homer2 proteins have been implicated in regulating addiction to cocaine in animal models (Szumlinski et al., 2004). A fine-tuned modulation of *Homer1a* expression has been associated to a number of mechanisms of adaptation to different environmental and pharmacological stressors: for instance, *Homer1a* overexpression in cortical structures may facilitate the ability to cope with stress (Szumlinski et al., 2006). Finally, *Homer1* gene variants have been associated with neuropsychiatric disorders such as psychosis in Parkinson disease (De Luca et al., 2009), major depression pathophysiology (Serchov et al., 2016), and response to lithium treatment (Benedetti et al., 2018).

#### *Homer1a* modulation by antipsychotics

Dopamine indirect agonists, such as cocaine or amphetamines, may induce *Homer1a* expression in striatum and nucleus accumbens with peculiar patterns of expression (Yano and Steiner, 2005; Zhang et al., 2007). Moreover, although the acute administration of cocaine may induce strong *Homer1a* expression in cortico-striatal circuits, these effects are abolished after 2 or 3 weeks of withdrawal (Ghasemzadeh et al., 2009), suggesting a crucial role of *Homer1a* in cocaine-mediated synaptic plasticity. Specific studies demonstrated that dopamine agonists-dependent *Homer1a* induction is regulated by selective activation of D1Rs but not D2Rs (Yamada et al., 2007). Recent findings by our group have demonstrated that this IEG may be differentially induced by antipsychotics, with a peculiar pattern of expression depending on the degree of D2R blockade by each compound and on the selective brain area in which each antipsychotic exerts its functions (Tomasetti et al., 2007). Haloperidol has been demonstrated to induce *Homer1a* expression specifically in dorso-lateral regions of caudate-putamen and in the core of the nucleus accumbens, a feature that is consisting with the propensity of this compound to provoke EPSE in humans at high dosages (Ambesi-Impiombato et al., 2007). By contrast, atypical antipsychotics (i.e., aripiprazole, clozapine, olanzapine, quetiapine, ziprasidone) preferentially induce *Homer1a* gene expression in ventro-medial regions of caudate-putamen and in the shell of the nucleus accumbens, wh1ich are brain regions implicated in the control of reward and motivated behavior (Tomasetti et al., 2007; Iasevoli et al., 2009). It is worthy to note that each antipsychotic compound has been described to induce a specific pattern of *Homer1a* expression that is tightly related to its degree of D2R blockade, being this latter essential in order to stimulate *Homer1a* induction (Iasevoli et al., 2011). *Homer1a* has been shown to be induced differentially also by the administration of the same antipsychotic at different doses: for example, it has been demonstrated that increasing doses of haloperidol not only increase the intensity (i.e., higher autoradiographic signal level) of gene expression in brain regions originally activated by the same drug at lower doses, but also induce the expression of the IEG in new brain regions (i.e., ventral caudate; De Bartolomeis et al., 2015a). Moreover, *Homer1a* modulation has been described in cortical areas only by antipsychotics that may impact serotonergic neurotransmission (Iasevoli et al., 2010a,b). Recent studies, indeed, demonstrated that cortical *Homer1a* induction by antipsychotics may resemble that by selective serotonergic agents, and when co-administered, haloperidol plus a selective serotonergic reuptake inhibitor antidepressant (SSRI, i.e., citalopram or escitalopram) may induce a pattern of *Homer1a* cortical expression tightly resembling the pattern by atypical antipsychotics (Dell'aversano et al., 2009; Serchov et al., 2016). Further evidence demonstrated that *Homer* genes may be involved also in synaptic rearrangements induced by combined mood-stabilizing/antipsychotic treatment (Tomasetti et al., 2011) and in switching between antipsychotics (De Bartolomeis et al., 2016). In sum, regarding the mechanism by which antipsychotics increase *Homer1a* and in turn may modify dendritic spine, D2Rs antagonism or partial agonism is the major candidate, possibly with a mechanism CRE-related, even if other pathways (i.e., ERK-related) can also be involved. *Homer1a* induction may have a pivotal role in remodeling the dendritic spine, modifying the availability of the constitutive isoform (*Homer1b*/*c*) that is involved in a transient spine increase that is eventually followed by more persistent modification by recruitment of other postsynaptic proteins such as PSD-95 (Meyer et al., 2014).

Altogether, these data confirm the role of *Homers* in the fine modulation of synaptic processes triggered by psychotropic drugs also when co-administered with antidepressant or mood stabilizers, posing the bases for further understanding the molecular correlates of real-world clinical psychopharmacology. The pattern of *Homer* inducible isoform expression may therefore provide a specific “fingerprint” profile of psychopharmacologic treatments, which could be a useful tool for elucidating glutamate-dopamine interactions putatively dysfunctional in schizophrenia pathophysiology.

### BDNF: neurotrophic control of synaptic plasticity by antipsychotic treatment

#### BDNF activity-dependent modulation and synaptic plasticity

BDNF belongs to a subfamily of neurotrophins that includes the nerve growth factor (NGF), the neurotrophin-3 (NT3), and the neurotrophins 4 and 5 (NT4/5). Several studies have demonstrated that neuronal activity, or in general stimuli that increase intracellular levels of Ca2+, may induce BDNF expression in neurons (Aicardi et al., 2004; Aid et al., 2007). Specifically, exon IV transcription seems to be directly controlled by neural activation (Chen et al., 2003). Moreover, the rapid activity-dependent increase in *BDNF* mRNA after a stimulus and its independence from the most common TFs (such as AP-1), have suggested that *BDNF* may be rather considered a “secreted IEG,” because of its immediate-early response fashion that does not involve new protein synthesis (Lauterborn et al., 1996; Xu et al., 2000; Gartner et al., 2006). The activity-dependent modulation of BDNF, as well as the BDNF-dependent master control of synaptic functions, has increased the attention on this molecule in synaptic plasticity. Several reports, indeed, demonstrated that BDNF plays a crucial role in both early and late phases of hippocampal LTP (Pang and Lu, 2004; Rex et al., 2006; Yano et al., 2006), as well as suggest a pivotal role of BDNF also in long-term memory processes (Lu et al., 2008; Waterhouse and Xu, 2009). *BDNF* exerts also a regulatory role on other IEGs expression: *Arc* has been found to be a key molecular effector of *BDNF* action in synaptic plasticity since its expression is necessary for stable LTP formation after BDNF levels increase in both *in vivo* and *in vitro* experiments (Messaoudi et al., 2007; Wibrand et al., 2012; Panja et al., 2014).

As in the case of the IEGs considered before, several studies reported a direct correlation between dopamine neurotransmission and *BDNF* functions. Indeed, *BDNF* null mice have been demonstrated to display a reduced number of dopaminergic neurons in the substantia nigra (Baquet et al., 2005). Moreover, *BDNF* is crucial for a correct D3Rs expression in nucleus accumbens, and thereby it seems involved in pathological conditions in which these receptors have been reported as dysfunctional, such as Parkinson's disease or antipsychotic-induced TD (Guillin et al., 2001; Zai et al., 2009).

#### BDNF, schizophrenia, and modulation by antipsychotics

Given the tight association of *BDNF* functions with activity-dependent dopamine-mediated synaptic plasticity, it is not surprising that several studies have highlighted the role of *BDNF* in the pathophysiology of neuropsychiatric disorders in which dopamine-glutamate interaction is dysfunctional, such as schizophrenia. The rs6265 single nucleotide polymorphism (SNP)—which leads to the Val66Met substitution at codon 66—has been reported to alter the activity-dependent trafficking and release of *BDNF* in neurons (Chen et al., 2004). This SNP has been associated with schizophrenia in Chinese and Caucasian populations (Hong et al., 2003; Neves-Pereira et al., 2005; Chen et al., 2006). Moreover, SNP homozygotes display reduced hippocampal cortex (Takahashi et al., 2008). In post-mortem studies, a decrease in BDNF levels in frontal cortices (Weickert et al., 2003) and an increase in hippocampus have been reported in schizophrenia patients (Iritani et al., 2003). Moreover, in the assessment of 825 patients for Positive and Negative Syndrome Scale in a single marker analysis, the *BDNF* rs10835210 mutant A allele was significantly associated with schizophrenia. Haplotype investigation detected higher frequencies of haplotypes with the mutant A allele of the rs10835210 in schizophrenia patients than in controls (Zhang et al., 2016). In addition, schizophrenia patients showed lower basal serum levels of BDNF as compared to healthy subjects (Grillo et al., 2007; Green et al., 2011; Fernandes and Chari, 2016).

Association studies have further suggested that *BDNF* could be involved in both susceptibility to schizophrenia and in clinical symptom severity. Regarding the role of *BDNF* in the onset and evolution of psychosis, it is of interest that the expression of the two forms of BDNF receptors (active TrkB-FL and inactiveTrkB-T1) in Peripheral Blood Monocyte Cells (PBMCs) of first episode psychotic patients showed modifications according to the trajectory of the disease, with TrkB-FL expression increasing by 1 year after diagnosis and TrkB-T1 expression decreasing. Notably, the TrkB-FL/TrkB-T1 ratio increased in the non-affective psychosis group only (Martinez-Cengotitabengoa et al., 2016). Multiple studies have assessed the effects of antipsychotic treatments on *BDNF* expression in preclinical models, as well as of BDNF serum levels in treated schizophrenia patients. Early studies demonstrated that the acute blockade of NMDA receptors may decrease *BDNF* expression in hippocampus and cortical areas, whereas it may increase its expression in limbic cortex (e.g., entorhinal cortex), and these effects may be not reversed by the administration of haloperidol (Castren et al., 1993). Further studies have confirmed the enhancement of *BDNF* expression in entorhinal cortex by NMDA receptor-blocking drugs (i.e., MK-801), these effects being contrasted by a pre-treatment with haloperidol or clozapine (Linden et al., 2000). However, the sole acute or chronic clozapine treatment did not affect *BDNF* mRNA levels (Linden et al., 2000). Starting from the assumption that antipsychotic treatment could be correlated to neurotrophic actions in brain areas affected in schizophrenia, Angelucci et al. (2000) reported that chronic treatment with haloperidol or risperidone may decrease *BDNF* expression in hippocampus, frontal and occipital cortices, also affecting TrkB expression in these areas.

Typical and atypical antipsychotics have differential impact on *BDNF* expression in distinct brain areas. Chronic haloperidol administration may strongly decrease *BDNF* hippocampal expression, whereas clozapine and olanzapine have been demonstrated to enhance *BDNF* expression in the same areas, probably due to 5HT2A receptor modulation by these atypical antipsychotics (Bai et al., 2003). Moreover, olanzapine has been successively demonstrated to normalize *BDNF* hippocampal levels that were reduced by MK-801 administration (Fumagalli et al., 2003). Switching from haloperidol or chlorpromazine to olanzapine, even after a prolonged treatment, may restore *BDNF* brain level that have been decreased by the previously administered typical antipsychotics (Parikh et al., 2004; Pillai et al., 2006).

Park et al. (2009) reported that ziprasidone, but not haloperidol, may attenuate the decrease in *BDNF* expression induced by immobilization stress in rats. Aripiprazole, a partial agonist at D2/D3Rs and a functional selective antipsychotic (De Bartolomeis et al., 2015b), has been shown to up-regulate *BDNF* compared to haloperidol in cell cultures (Park et al., 2009). In early clinical studies, clozapine-treated schizophrenia patients showed higher serum BDNF levels than risperidone-treated patients (Tan et al., 2005). With regard to clinical translation it has been demonstrated that the increase in BDNF serum levels in olanzapine-treated schizophrenia patients may directly correlate with the progressive reduction in positive symptoms (Gonzalez-Pinto et al., 2010).

Regarding the role of BDNF in dendritic spines modulation, is remarkable that multiple lines of evidence point to a brain and cell region specificity of BDNF action.

Specifically, has been shown that BDNF increase in cortical regions may reduce the density of dendritic spines of pyramidal neurons, whereas an increase has been reported for hippocampal pyramidal neurons (Alonso et al., 2004). This finding, considered in the light of antipsychotics modulation of BDNF, could represent a significant morphological underpinning of the association between antipsychotics and changes in brain architecture.

It is questioned if a common ERK-dependent mechanism is involved in the opposite changes observed in cortex and hippocampus.

In summary, the study in preclinical and clinical settings of *BDNF* response to antipsychotics may help to provide further information on the differential impact of typical vs. atypical antipsychotics on neurons survival and neurogenesis, as well as on putative neurodegenerative mechanisms of dopaminergic systems involved in the pathophysiology of schizophrenia. *BDNF* role as an immediate-early-like gene, TF, and growth factor makes this molecule an exceptional candidate for the investigation of long-term antipsychotic effects on brain structure and function, and the study of the regulation of its expression could provide a molecular tool to predict clinical outcomes of antipsychotic response (Nandra and Agius, 2012).

## IEGs related to glutamate dependent plasticity: antipsychotic treatment effects on *Npas* 4 and *Narp* expression

*Npas4* and *Narp* are IEGs related to glutamate and γ-aminobutyric acid (GABA) neurotransmission, whose implication in schizophrenia pathophysiology and treatment to date has been explored only by few clinical and preclinical studies.

### *Npas4*: an IEG selectively induced by neuronal activation

*Npas4* is a TF that belongs to the basic helix-loop-helix-PAS protein family (Moser et al., 2004; Shamloo et al., 2006) that is transcribed in response to excitatory synaptic activity induced in both excitatory and inhibitory neurons. *Npas4* is expressed almost exclusively in neurons, it is activated selectively by neuronal activity, and has been demonstrated to control directly the expression of a large number of activity-dependent genes (Coutellier et al., 2012). Recent evidence shows that *Npas4* has a regulatory function on the expression of multiple cortical GABAergic markers and that animals null for *Npas4* show a decrease in GAD67 and parvalbumin, which can be reverted after the administration of valproic acid (Shepard et al., 2017). Moreover, *Npas4* has been involved in stress response and linked to the onset of resistance to L-acetyl carnitine in mice (Bigio et al., 2016). Particularly, this IEG has been shown to regulate the balance between neuronal excitation and inhibition by contributing to the maintenance of the inhibitory pathways. This balance is believed to be pivotal for processing sensory information and for cognitive functioning, while an imbalance between inhibitory and excitatory synapses has been associated with multiple developmental disorders such as schizophrenia. Not surprisingly, it has been recently published the first study that investigated *Npas4* expression after antipsychotic administration in rodents. Indeed, the IEG has been demonstrated to be down-regulated acutely, but not chronically, in the cortex by the novel antipsychotic lurasidone (10 mg/kg) at the dose demonstrated to be effective in animal models of schizophrenia (Luoni et al., 2014b).

### *Narp*: an IEG secreted by pyramidal neurons

Narp (Neuron activated regulated pentatraxin) is an AMPA receptor binding protein with the peculiarity to be secreted by pyramidal neurons onto parvalbumin interneurons and whose gene is rapidly transcribed and regulated by physiological synaptic activity (O'brien et al., 1999, 2002; Chang et al., 2010; Lee et al., 2017). Functional studies suggest that Narp promotes neuronal migration and dendritic outgrowth with a potency comparable to neurotrophins and growth factors (Tsui et al., 1996; Doyle et al., 2010). *Narp* is a direct transcriptional target of BDNF. Intriguingly, acute BDNF withdrawal may promote downregulation of *Narp*, whereas transcription of *Narp* is greatly enhanced by BDNF (Mariga et al., 2015). Furthermore, it has been demonstrated that BDNF directly regulates *Narp* to mediate glutamatergic transmission and mossy fiber plasticity (Mariga et al., 2015). Hence, *Narp* serves as a significant epistatic target of BDNF to regulate synaptic plasticity during periods of dynamic activity. Recently, a close association between *Narp* expression and schizophrenia pathophysiology has been suggested. In a post-mortem study conducted on brain specimens (*n* = 206) from schizophrenia, bipolar disorder, and major depressive disorder patients, *Narp* transcript expression was measured at the level of the dorsolateral prefrontal cortex. A significant 25% reduction of *Narp* mRNA expression was detected in schizophrenia patients compared to normal controls (Kimoto et al., 2015). Moreover, as in the case of *Npas4*, the expression of *Narp* after antipsychotic administration has been explored only in one study to date. Indeed, the IEG has been demonstrated to be differentially regulated by haloperidol (1 mg/kg i.p.) and clozapine (20 mg/kg i.p.) in cortical and subcortical rat brain regions Particularly, it has been shown that clozapine causes a specific decrease of *Narp* in the striatum (Robbins et al., 2008).

In summary, *Npas4* and *Narp* share peculiar IEGs characteristics, the first one being expressed exclusively in neurons, and the second one being specifically secreted onto pyramidal cells. Both are deeply linked to glutamatergic and GABAergic functions. All the above-mentioned features make these early genes potential great players both in schizophrenia pathophysiology and in antipsychotic mechanisms of action at the intracellular level. Therefore, further studies are required in order to better clarify their putative specific roles in the disease development and treatment strategies.

## Conclusions

### IEGs may set the scenario for acute and long-term changes induced by antipsychotics

Antipsychotic agents are the mainstay of treatment in schizophrenia and in other psychotic disorders. However, despite half a century of research, their ultimate molecular actions and the neurobiological mechanisms beyond D2R occupancy are still elusive. *In vivo* human studies have shown that volumetric and functional changes may occur after chronic antipsychotic treatment and that some changes may be detected even after acute antipsychotic administration. Notably, schizophrenia has been considered a disease of synaptic plasticity and of dendritic spines (Penzes et al., 2011), and it is conceivable that antipsychotics exert their action by triggering a complex set of structural and functional modifications, also at the level of dendritic spines (De Bartolomeis et al., 2014).

Tracking down the initial wave of molecular changes from multiple receptor interactions to dendritic spines modifications is a challenging task. In this context, IEGs may represent ideal candidates to explore how antipsychotics may set the scenario for acute and chronic re-arrangements of genes expression at the synapse.

This essential role of IEGs may be justified by multiple reasons:

1. IEGs control the early molecular processes of rapid synaptic plasticity induced by antipsychotics, which do not always require *de novo* protein synthesis.
2. IEGs are pleiotropic molecules that, beyond the common feature of being activated rapidly by diversified stimuli, are characterized by differential and specific functions spanning from transcription modulation (i.e., *c-fos*), to neurotrophic action (i.e., *BDNF*), to regulation of scaffolding proteins at the PSD (i.e., *Homer1a*), all of which have been demonstrated to be induced by antipsychotics.
3. IEGs are strongly involved in complex higher functions affected by antipsychotics in key brain regions implicated in memory and learning (i.e., prefrontal cortex), as well as in reward and volition (i.e., limbic circuits), all of which have been demonstrated to be altered in schizophrenia (Leber et al., 2017).
4. IEGs expression may vary in response to antipsychotics, with a differential level of expression related to the receptor-binding profile (i.e., antipsychotics with prevalent dopaminergic activity vs. antipsychotics with more complex receptor profiles), to the dose, and to the duration of the treatment.
5. Different antipsychotic compounds have been demonstrated to induce specific patterns of IEGs expression in peculiar brain areas implicated in schizophrenia pathophysiology (Matosin et al., 2016).

### Future steps toward a further clarification of IEGs modulation by antipsychotics

Despite the lot of findings that highlight the complex modulation of IEGs by antipsychotics, the road for taking full advantage of this class of molecules in better understanding antipsychotics mechanisms of action is still long-lasting.

Here are listed few points that may make the issue progress:

1. It would be relevant to develop a research strategy to track down IEGs as putative blood biomarkers for antipsychotics activity under a diagnostic and therapeutic (“theranostic”) approach. A recent preclinical investigation has tried to apply this strategy to antidepressant therapy starting from IEGs comparative analysis in brain regions and blood (Waller et al., 2017).
2. IEGs monitoring after acute or continuous antipsychotic treatments should be carried out in *in vivo* models, in order to better understand the role of this class of molecules in the mechanisms of action of antipsychotics. For example, multiphoton imaging of IEGs signals in cortical circuits has already been successfully used to get information on *Egr1* expression after the exposure to a novel context (Xie et al., 2014).
3. The correlation among IEGs induction, antipsychotics, and epigenetic modulation is an attractive new scenario worth to be explored, which is based on the demonstrated modulation of the methylome by antipsychotics, on the recent findings on epigenetic control of IEGs in brain, and on the relevance of the contextual epigenetic-based mechanisms regulating brain higher functions that are involved in psychosis pathophysiology (Saunderson et al., 2016; Srivas and Thakur, 2016).
4. Under a translational perspective, more pharmacogenomic studies and brain post-mortem imaging investigations evaluating antipsychotics-dependent IEGs induction are needed to address in humans the findings that have already been described in animal models.
5. Finally, and again from a translational perspective, it will be important to start considering the “druggable” potential of some IEGs such as *Homer1a*, in order to search for new putative therapeutic strategies that can reach the core of the synapse and eventually correct the alterations linked to aberrant synaptic plasticity (Dev, 2004; Menard et al., 2015).

In sum, IEGs modulation by antipsychotics may provide a key tool to better understand the brain topography of antipsychotic action, the multiple pathways involved in the acute effects of these therapeutics beyond receptor interactions, as well as the molecular background for long-term changes of synaptic architecture promoted by chronic antipsychotic exposure.

## Author contributions

AdB conceived the rationale and the structure of the review, wrote the manuscript and revised the literature search; EB analyzed the literature, contributed to write some sections of the second draft and to revise the final draft; FI, CT, GL, and FM revised and made contribution on the final draft; RR made the initial literature research and contribute to write partially the first draft of the manuscript; CT made the literature search, contributed to write the different drafts of the article.

### Conflict of interest statement

The authors declare that the research was conducted in the absence of any commercial or financial relationships that could be construed as a potential conflict of interest.
